# An outbreak of type B botulism in southern Viet Nam, 2020

**DOI:** 10.5365/wpsar.2022.13.1.887

**Published:** 2022-01-06

**Authors:** Tinh Huu Ho, Ha Phan Ai Nguyen, Nhan Dinh Trong Le, Phuong Hoai Hoang, Ninh Thi Ha, Chinh Van Dang

**Affiliations:** a Institute of Public Health, Ministry of Health, Ho Chi Minh City, Viet Nam.

## Abstract

**Objective:**

To investigate the cause of a botulism outbreak in several provinces in Viet Nam in 2020.

**Methods:**

An initial investigation was conducted to confirm the outbreak and to form hypotheses about the potential causes, followed by a case–control assessment of the plausible causative food item. Collected food samples were tested to identify the pathogen, and mouse bioassays were performed. Control measures were introduced to stop the outbreak and to prevent similar events in the future.

**Results:**

Twelve people in six southern provinces of Viet Nam were identified as having symptoms of botulism, of whom 11 were in critical condition requiring breathing support. A history of foods eaten in the 4 days before illness onset indicated that all the cases had eaten a tinned vegetarian pâté, and a case–control assessment showed that this was significantly associated with the outbreak, with an odds ratio of 35.2 (95% confidence interval: 3.4–¥). *Clostridium botulinum* type B was detected in three of eight pâté samples collected from the houses of cases. In the mouse bioassay for the toxicity of the pâté samples, all the mice died with clinical symptoms of botulism.

**Discussion:**

A tinned vegetarian pâté was the plausible cause of a botulism outbreak in Viet Nam in 2020. Revision of food safety regulations to improve quality control of tinned foods to prevent future outbreaks is recommended.

Botulism is a life-threatening condition caused by botulinal neurotoxins (BoNTs). The typical symptoms are neurological, including blurred vision, slurred speech, difficulty swallowing and muscle weakness. (*1*) The mortality rate may be up to 60% without adequate medical intervention. (*1*) BoNTs are produced by *Clostridium botulinum*, a Gram-positive, rod-shaped, anaerobic, spore-forming, motile bacterium. (*2*) Of the eight types of BoNT (A–H), A, B, E and F are associated with human botulism. (*1*) *C. botulinum* spores grow and produce toxins in foods in an anaerobic, non-acidic environment with low sugar and salt. The spores are highly resistant to heat (several hours at 100 °C), desiccation, ultraviolet light and alcohol. (*1*) *C. botulinum* is present in the environment, with type A or B spores being found primarily in terrestrial vegetables and type E commonly found in fish and aquatic products. (*3*)

Several outbreaks of botulism have been reported globally, due to consumption of a wide range of foods. In Egypt, a type E botulism outbreak was reported in 1991 in 91 patients, with 19 fatalities, related to consumption of a fermented grey mullet fish (faseikh). (*4*) In Finland, an outbreak of BoNT type E in 1997 was linked to consumption of hot-smoked Canadian whitefish. (*5*) Tinned bamboo shoots were found to be the cause of three outbreaks of botulism in Thailand, comprising nine cases in 1998, (*6*) 19 cases in 1997–1998 (*7*) and up to 209 cases in 2006. (*8*) In China, Taiwan (China), two outbreaks have been recorded, one caused by type A botulism in nine patients who consumed preserved peanuts in 1986 (*9*) and another caused by type B botulism in five cases related to consumption of fermented food in 2006. (*10*) In China, two type A BoNT outbreaks were caused by consumption of smoked ribs by two patients in 2013 (*11*) and of vacuum-packaged salted fish and ham in four cases in 2021. (*12*) Liquid herbal tea was found to be the main source of a type A botulism outbreak in two elderly people in the United States in 2017. (*13*)

No outbreaks of botulism had been reported in Viet Nam before the recent outbreak in the southern provinces in July 2020. (*14*) An initial case series from this outbreak, which comprised the first six cases in a hospital in Ho Chi Minh City, linked cases to consumption of a tinned vegetarian pâté. In addition, the Institute of Public Health (IPH) within the Ministry of Health, located in Ho Chi Minh City, received notification of several suspected cases from this and two other hospitals via a hotline. The aim of this study was therefore to investigate the source of the botulism outbreak by examining food consumption histories and pathogens from collected samples to provide information for preventing future foodborne outbreaks.

## Methods

The investigation was conducted in three stages: 1) an initial investigation to confirm the outbreak; 2) an epidemiological investigation to identify foods possibly implicated in the outbreak, including a case–control assessment of the implicated food product; and 3) a laboratory investigation to identify the pathogen. Environmental studies and investigations at food facilities related to the outbreak were conducted by a different institution, and the results were not shared with our investigation team.

### Initial investigation

At the end of July 2020, seven patients with severe neurological symptoms were transferred from four provincial hospitals to two central hospitals in Ho Chi Minh City. Initial diagnosis of the six initial cases suggested a potential outbreak of botulism intoxication. (*14*) A full investigation of all cases in the southern provinces was conducted by the rapid outbreak response team of the IPH. Although cases were also recorded in northern provinces, they were not included in this investigation because of limited resources.

Details of the cases in all hospitals in the southern provinces were collected to confirm whether an outbreak of food poisoning had occurred. The team examined hospital case reports, consulted physicians who treated the patients and interviewed the patients or their caregivers. To avoid missing new cases, the IPH also notified all central hospitals in Ho Chi Minh City to report all suspected cases to IPH between 1 August and 30 September 2020.

The case definition used for the outbreak was any individual admitted to any central hospital in Ho Chi Minh City between July and September 2020 who was diagnosed with botulism intoxication or any individual with three or more of the following symptoms: limb weakness, bilateral ptosis, dysphagia, difficulty breathing, dysarthria, descending paralysis and double and blurred vision, during the same period who was not admitted to hospital. Age, sex, symptoms, date of onset and history of foods consumed during the 4 days before the date of onset were recorded for each patient.

The incubation period was calculated as the time between consumption of the pâté and the onset of symptoms. For patients who ate the pâté more than once during the 4 days before onset of symptoms, the incubation period was calculated as the time between the first and last consumption of the pâté and the onset of symptoms.

### Case–control assessment

After the initial investigation, a case–control assessment was conducted to confirm epidemiologically that the implicated food item was the source of the outbreak. The cases were those from the initial investigation, and controls were defined as people who had shared at least one meal with a case in the 4 days before the onset of illness in that case, who showed none of the above symptoms of botulism intoxication.

A structured questionnaire was used to interview the cases and controls, which included age, sex and history of food consumption (including names of foods and quantity ingested) within 4 days of the onset of any symptom. Controls were asked only about their exposure to the implicated food. Odds ratios (ORs) were calculated by exact logistic regression, and *P*-values were calculated for exposure to the implicated food only with Fisher’s exact test.

### Laboratory investigation

Vegetarian pâté samples from opened tins that had been consumed by patients within 4 days of symptom onset and samples from three unopened tins were collected from household members and sent to the IPH for examination. Patient specimens (faeces and stomach fluid) collected in hospital at admission were also examined.

Testing for *C. botulinum* and botulinal toxin was conducted according to standard methods. (*15*) Briefly,  1 g of pâté sample was added to 15 mL of cooked meat broth in a tube (Becton Dickinson, Sparks, MD, USA). After 5 days of incubation at 35 °C, enrichment cultures were examined for turbidity, gas production, digestion of meat particles, odour and Gram stain. The enrichment culture was also inoculated onto anaerobic egg yolk agar (HiMedia, India) and incubated at 35 °C for 48 hours. A single pearly zone colony was selected and inoculated into trypticase peptone glucose yeast extract broth (HiMedia). After incubation for 5 days at 26 °C, the culture in broth was used for further detection of toxin in a mouse bioassay. The culture was diluted 1:5, 1:10 and 1:100 in gelatin phosphate buffer (HiMedia), and mice weighing 15–20 g were injected intraperitoneally with 0.5 mL of each dilution of test sample. All the mice were observed periodically for symptoms of botulism for 48 hours. Typically, signs of botulism in mice begin within the first 24 hours with ruffling of fur, followed in sequence by laboured breathing, weakness of limbs and total paralysis with gasping for breath, followed by death due to respiratory failure. *C. botulinum* isolates carrying botulinum neurotoxin A, B, E and F genes were identified in a polymerase chain reaction (PCR) assay, as reported previously. (*16*)

## Results

### Initial investigation

Twelve cases of botulism were linked to the outbreak between 24 July and 15 September 2020, 11 of which were treated in intensive care units in three central hospitals; the other case was not admitted to hospital. The cases ranged in age from 20 to 64 years (median, 38 years). Eight of the 12 were female and reported eating a vegetarian diet. The cases were from six southern provinces, with five cases from two families (two cases in Khanh Hoa province and three in Long An province), three cases among roommates in Dong Nai province and four unlinked cases from Vung Tau (*n* = 2), Ho Chi Minh City and Binh Duong (**Table 1**).

**Table 1:** Characteristics of cases and controls in the botulism outbreak investigation, southern Viet Nam, 2020

Characteristic	Cases (*n* = 12)		Controls (*n* = 9)	
	*n*	%	*n*	%
Age (years), median (min–max)	38 (20–64)		27 (18–56)	
Sex				
Female	8	67	8	89
Male	4	33	1	11
Vegetarian diet	8	67	3	33
Home province				
Long An	3	25	0	0
Dong Nai	3	25	2	22
Khanh Hoa	2	17	0	0
Vung Tau	2	17	5	56
Ho Chi Minh City	1	8	2	22
Binh Duong	1	8	0	0
Symptoms				
Limb weakness	10	83	-	-
Bilateral ptosis	9	75	-	-
Dysarthria	8	67	-	-
Vomiting	6	50	-	-
Difficulty breathing	5	42	-	-
Hospitalized	11	92	-	-
Required ventilator	11	92	-	-
Relationship to case patients				
Roommate	-	-	2	22
Child	-	-	3	33
Spouse	-	-	2	22
Parent or grandparent	-	-	2	22

The three most common symptoms were limb weakness (10/12), bilateral ptosis (9/12) and dysarthria (8/12). All 11 hospitalized patients required ventilator support. No deaths were reported (**Table 1**). The four most serious cases were treated with botulinum antitoxins provided by the World Health Organization; however, we were unable to evaluate the effectiveness of botulinum antitoxin treatment.

**Fig. 1:**
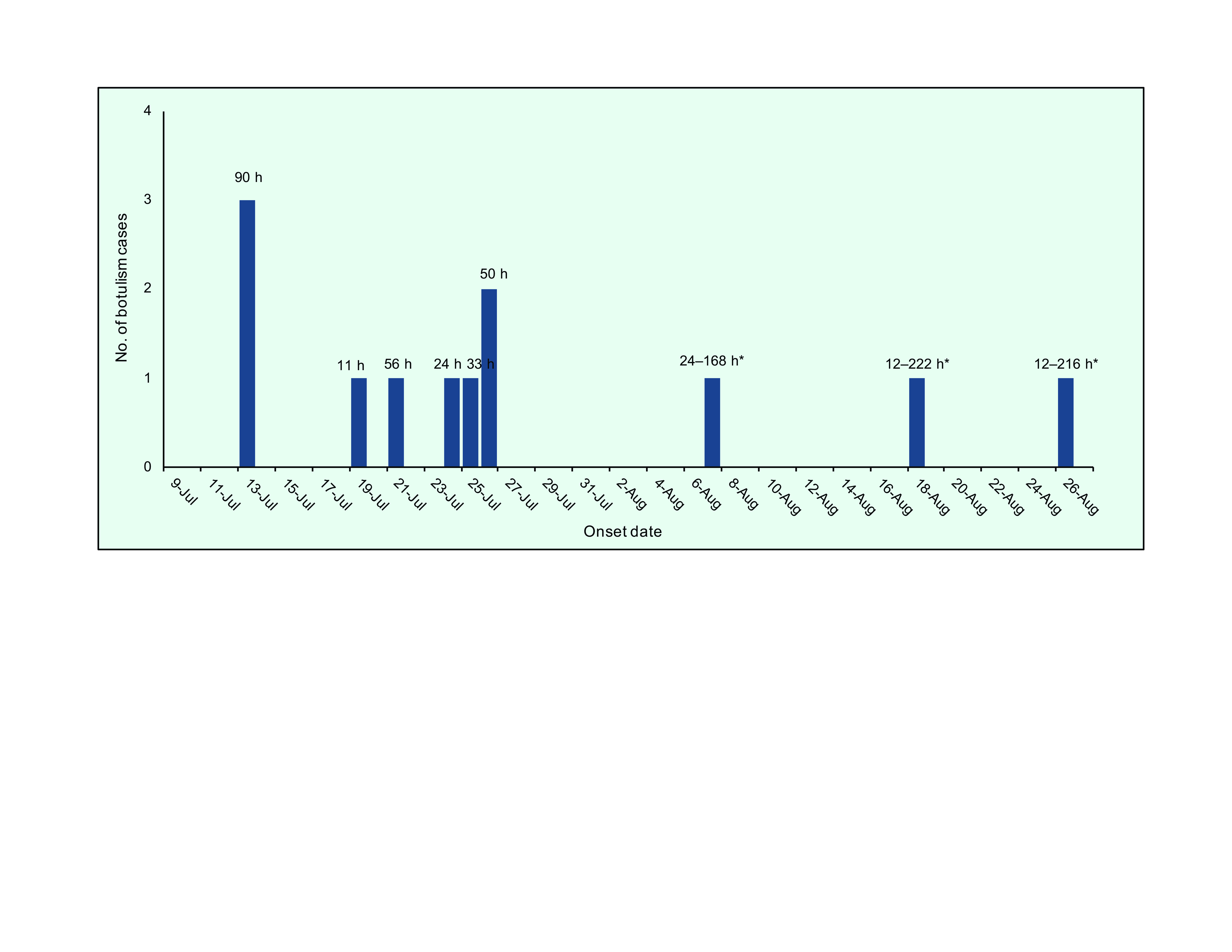
Epidemic curve and incubation period after consumption of the implicated food of 12 cases of botulism, southern Viet Nam, 2020

All 12 cases reported having eaten the same brand of tinned vegetarian pâté in the 4 days before symptom onset. All but one case reported having eaten at least 1.5 spoonfuls. The ingredients of the pâté were mushrooms (such as shiitake, wood ear, chicken drumstick, oatmeal and straw mushrooms), soya bean and nuts (cashew, almond and walnut). All other foods reported in the food histories were consumed by 17–42% of cases (**Table 2**). The incubation period ranged from 11 to 222 hours (median, 73 hours) (**Fig. 1**).

[**insert** Figure 1]

**Table 2:** Food consumption of 12 cases of botulism in the 4 days before onset of symptoms – southern Viet Nam, 2020

Food	*n*	%
Vegetarian pâté	12	100
Bread	5	42
Steamed rice	5	42
Pickled vegetables	5	42
Vegetable soup	3	25
Steamed vegetables	3	25
Tofu	3	25
Sweet taro	3	25
Potato	3	25
Bamboo shoot	3	25
Noodle	2	17
Snail	2	17
Cumulative intake of vegetarian pâté (spoonfuls)		
£ 1	1	8
1.5	6	50
2	3	25
> 2	2	17

### Case–control assessment

All 12 cases and nine controls were included in the case–control assessment of the implicated food product. As most of the cases were vegetarians, their meals were usually different from those of other household members; thus, it was difficult to find controls who had shared meals with the cases. The nine controls identified had all shared a meal with the cases in the 4 days before symptom onset and were relatives (children, spouses, parents or grandparents) or roommates (**Table 3**). Controls were not obtained for six cases who ate meals different from those of other household members.

**Table 3:** Odds ratios (ORs) and 95% confidence intervals (CIs) for case and control consumption of vegetarian pâté – southern Viet Nam, 2020

Pâté consumption	Case (*n* = 12)	Control (*n* = 9)	OR (95% CI)
Pâté eaten			
Yes	12	2	35.2 (3.4–¥)
No	0	7	-
Cumulative amount of pâté eaten			
> 1 coffee spoon	11	0	12.5 (0.84–¥)
£ 1 coffee spoon	1	2	-

As the only food item consumed by all cases was a tinned vegetarian pâté, the case–control analysis included only the pâté. Only two controls reported having eaten the pâté, giving an OR of 35.2 (95% confidence interval [CI]: 3.4–¥). The OR for consuming more than one spoonful of pâté was 12.5 (95% CI: 0.84–¥); however, this association was not statistically significant.

### Laboratory investigation

Eight pâté samples, three from unopened tins and five from opened tins and consumed by the cases, and specimens from four patients were collected (**Table 4**). Three of five opened pâté samples were positive for BoNT type B *C. botulinum* by PCR test. None of the unopened tinned pâté samples was positive for BoNT. In the mouse bioassay, all mice died with clinical symptoms of botulism after exposure to the three pâté samples positive for *C. botulinum*. The four specimens from cases were all negative for *C. botulinum*. Six cases had consumed pâté from the three opened tins that were positive for *C. botulinum*: three in Dong Nai province, one in Ho Chi Minh City and two in Khanh Hoa province.

**Table 4:** Results of testing of food and patient samples – southern Viet Nam, 2020

Substrate	Type of sample	Culture	PCR	Toxicity testing in mice
Vegetarian pâté 1	2 jars, 450 g (unopened)	-	N/A	N/A
Vegetarian pâté 2	1 jar, 200 g (unopened)	-	N/A	N/A
Vegetarian pâté 3	1 jar, 450 g (unopened)	-	N/A	N/A
Vegetarian pâté 4	1 jar, 200 g (used)	+	Type B	Died, typical symptoms
Vegetarian pâté 5	1 jar, 200 g (used)	+	Type B	Died, typical symptoms
Vegetarian pâté 6	1 jar (used)	-	N/A	N/A
Vegetarian pâté 7	1 jar (used)	-	N/A	N/A
Vegetarian pâté 8	1 jar (used)	+	Type B	Died, typical symptoms
Gastric fluid	-	-	N/A	N/A
Gastric fluid	-	-	N/A	N/A
Faeces	-	-	N/A	N/A
Gastric fluid	-	-	N/A	N/A

## Discussion

This outbreak of botulism comprised 12 cases in six southern provinces of Viet Nam in 2020. No deaths were recorded, probably due to timely treatment of all patients and to administration of botulinum antitoxins provided by the World Health Organization to the four most serious cases. In addition, the strain identified was BoNT type B, which is less likely to be fatal than other types, such as BoNT types A and E. (*1*)

The most plausible source of this outbreak was a tinned vegetarian pâté. All the cases reported having eaten the same brand of the pâté, and the case–control assessment found that consumption of the pâté was significantly associated with the outbreak, with an OR of 35.2. The incubation period was estimated to be 11–222 hours (median, 73 hours), consistent with foodborne botulism, which can occur between 2 hours to 8 days after exposure (usually 12–72 hours). (*1*) In the laboratory investigation, positive results for *C. botulinum* type B were found in three opened pâté tins. The finding that all four patient specimens were negative for *C. botulinum* may be explained by the fact that the adult gastrointestinal tract is not a natural habitat for *C. botulinum*. Detection of BoNTs in patient specimens was also hindered by lack of reagents for additional testing.

After identification of the contaminated pâté as the cause of this outbreak, the information was widely spread through all media channels, and the population was advised not to eat the product; furthermore, all stocks of the product were recalled. The outbreak was successfully controlled, as no additional cases were identified at the national level.

As the pâté implicated in this outbreak was distributed nationally, cases occurred throughout the country. The ingredients of the pâté – mushrooms, soya beans and nuts – are common environments for C. botulism types A and B. (*1*) A limitation of this investigation was the inability to determine at which step(s) of the pâté production process the contamination was introduced, as the environmental investigation was conducted by another team, and our investigation team could not inspect the canning company. Foods may be contaminated with *C. botulinum* at all steps of the production process, including cultivation, harvesting, processing and after processing. Contamination often occurs during growth in an environment with a high incidence of spores. (*17*) *C. botulinum* is common in soil and organic fertilizers, usually at low concentrations. (*1*) In a survey in China, up to 25 000 *C. botulinum* spores were found per kg of soil and 2100 type B spores per kg of mushrooms. (*17*)

As *C. botulinum* is ubiquitous in the environment, viable *C. botulinum* or *C. botulinum* spores can occur in food. Botulism occurs only when *C. botulinum* in foods has enough nutritional requirements and anaerobic conditions for growth and production of toxins, such as in tinned foods. Although the optimum temperature for the growth and production of toxins is 35–40 °C, BoNTs can be produced at 3 °C. (*1*, *18*) The normal temperature in a refrigerator compartment, which is usually set at  4.4 °C (40 °F) or less, may not be low enough to inhibit the growth of *C. botulinum*. The growth of *C. botulinum* in tinned foods can nevertheless be controlled by several methods, such as low pH, low water activity, high salt concentration and other food preservatives. (*16*)

The limitations of this outbreak investigation are that it was restricted to the southern provinces of Viet Nam and did not include cases linked to consumption of the vegetarian pâté in the northern provinces Lack of access to the results of the environmental investigation also limited our study. The availability of reagent kits for detection of BoNTs has been a challenge for all laboratories in Viet Nam. In this study, we attempted to cultivate *C. botulinum* from samples and then used PCR to determine BoNT type, in addition to performing mouse bioassays. Another limitation of the response to this outbreak is that botulism is not covered in the national surveillance system, and no outbreaks of botulism had been seen previously.

In conclusion, this outbreak highlights the risk of botulism from tinned foods, especially once they are opened. Strengthening of regulation of the production of processed foods and public education on food safety at home are recommended to prevent future foodborne outbreaks. Although botulism is rare, preparation of reagent kits for early detection of BoNTs and a standard response protocol to ensure prompt investigation and implementation of control measures should be considered.
